# Curcumin: A Golden Approach to Healthy Aging: A Systematic Review of the Evidence

**DOI:** 10.3390/nu16162721

**Published:** 2024-08-15

**Authors:** Yandra Cervelim Nunes, Nathalia M. Mendes, Enzo Pereira de Lima, Amanda Chabrour Chehadi, Caroline Barbalho Lamas, Jesselina F. S. Haber, Manoela dos Santos Bueno, Adriano Cressoni Araújo, Vitor C. Strozze Catharin, Claudia Rucco P. Detregiachi, Lucas Fornari Laurindo, Masaru Tanaka, Sandra Maria Barbalho, Maria José Sanches Marin

**Affiliations:** 1 Faculdade de Medicina de Marília (FAMEMA), Marília 17519-030, SP, Brazil; yandracervelin@hotmail.com (Y.C.N.); lucasffffor@gmail.com (L.F.L.); 2 Department of Biochemistry and Pharmacology, School of Medicine, University of Marília (UNIMAR), Marília 17525-902, SP, Brazil; nathalia.m.machado@outlook.com (N.M.M.); enzopereiralima@outlook.com (E.P.d.L.); amandachehadi@outlook.com (A.C.C.); hjesselina@gmail.com (J.F.S.H.); manuelasantosbueno@outlook.com (M.d.S.B.); adriano_cressoni_araujo@outlook.com (A.C.A.); 3 Department of Gerontology, School of Gerontology, Universidade Federal de São Carlos (UFSCar), São Carlos 13565-905, SP, Brazil; carolinelamas@estudante.ufscar.br; 4 Postgraduate Program in Structural and Functional Interactions in Rehabilitation, University of Marília (UNIMAR), Marília 17525-902, SP, Brazil; vitorcavallaristrozzecatharin@gmail.com (V.C.S.C.); claudiaprucco@gmail.com (C.R.P.D.); 5 Danube Neuroscience Research Laboratory, HUN-REN-SZTE Neuroscience Research Group, Hungarian Research Network, University of Szeged (HUN-REN-SZTE), Tisza Lajos krt. 113, H-6725 Szeged, Hungary; 6 Department of Biochemistry and Nutrition, School of Food and Technology of Marília (FATEC), Marília 17500-000, SP, Brazil; 7 Research Coordination, Hospital Beneficente (HBU), University of Marília (UNIMAR), Marília 17525-160, SP, Brazil

**Keywords:** *Curcuma longa*, curcumin, aging, inflammation, oxidative stress, frailty, neurodegeneration, sarcopenia, cardiovascular diseases, clinical trials

## Abstract

Aging-related disorders pose significant challenges due to their complex interplay of physiological and metabolic factors, including inflammation, oxidative stress, and mitochondrial dysfunction. Curcumin, a natural compound with potent antioxidant and anti-inflammatory properties, has emerged as a promising candidate for mitigating these age-related processes. However, gaps in understanding the precise mechanisms of curcumin’s effects and the optimal dosages for different conditions necessitate further investigation. This systematic review synthesizes current evidence on curcumin’s potential in addressing age-related disorders, emphasizing its impact on cognitive function, neurodegeneration, and muscle health in older adults. By evaluating the safety, efficacy, and mechanisms of action of curcumin supplementation, this review aims to provide insights into its therapeutic potential for promoting healthy aging. A systematic search across three databases using specific keywords yielded 2256 documents, leading to the selection of 15 clinical trials for synthesis. Here, we highlight the promising potential of curcumin as a multifaceted therapeutic agent in combating age-related disorders. The findings of this review suggest that curcumin could offer a natural and effective approach to enhancing the quality of life of aging individuals. Further research and well-designed clinical trials are essential to validate these findings and optimize the use of curcumin in personalized medicine approaches for age-related conditions.

## 1. Introduction

Population aging is a global trend that brings medical, social, and economic challenges [1,2,3,4]. Aging is the result of a diversity of molecular and cellular damages accumulated in the body over the years. It is a natural, gradual, complex, and irreversible process, accompanied by systemic changes that cause a reduction in functionality and increase the risk of age-associated diseases that eventually result in death. Some characteristics of aging have been associated with loss of proteostasis, dysregulated nutrient sensing, increase in the oxidative processes, inflammation, telomere attrition, genomic instability, stem cell exhaustion, cellular senescence, impaired intercellular communication, and mitochondrial dysfunction [5,6,7,8,9].

As life expectancy increases, an increase in the incidence of chronic diseases is observed, including neurodegenerative diseases (NDs) such as Alzheimer’s disease (AD) and Parkinson’s disease (PD) [10], memory impairment [11], cognitive dysfunction [12,13], type 2 diabetes (DM2) [14], cardiovascular disease [15], cancer [16], and musculoskeletal disorders such as sarcopenia [4,17]. These conditions pose a substantial challenge to healthcare systems around the world due to their significant impact on the quality of life of the elderly, healthcare costs, and the global burden of disease [18,19].

**Figure 1:**
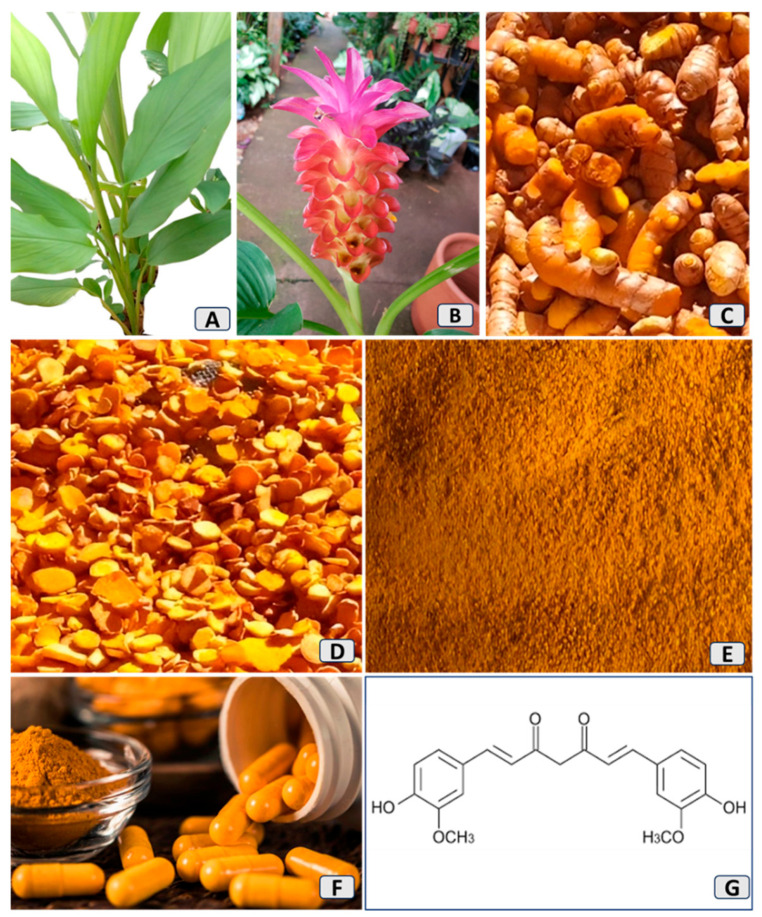
The main parts of the *Curcuma longa* plant. (**A**): leaves; (**B**): flower; (**C**): rhizomes; (**D**): sliced rhizomes; (**E**): rhizome powder; (**F**): pharmaceutical formulation, and (**G**): curcumin molecular structure.

Natural bioactive compounds have been used for thousands of years as adjuvants in therapeutic practice due to their efficiency, low cost, few side effects, and easy use [20,21,22]. *Curcuma longa*, known as turmeric or saffron, has received prominence among countless plants. It is an herbaceous and rhizomatous plant (Figure 1) from the ginger family, *Zingiberaceae*, and is native to Southeast Asia and India. For centuries, it has been used in cooking and as medicine [23,24,25,26,27].

Its main phenolic bioactive compounds are curcumin (~77%), demethoxycurcumin (~17–19%), and bisdemethoxycurcumin (~4%), which are extracted from the rhizomes [20,28,29], and can promote countless therapeutic actions by virtue of their anti-inflammatory and antioxidant potential, as shown in numerous clinical and preclinical studies [30,31,32,33]. It is a potential anticancer agent with the ability to modulate the main pathways involved in inflammation processes, oxidative stress (OS), carcinogenesis, autophagy, apoptosis, and cardiovascular and neurodegenerative conditions [23,34,35,36]. Furthermore, it has antidiabetic and antiobesogenic effects, provides cardiovascular protection, and can also reduce sarcopenic processes. Studies in hypertensive rats also show that it can prevent hypertension development by improving vascular remodeling and endothelial dysfunction [37,38,39,40].

Curcumin has shown positive effects in delaying the aging process and delaying age-related changes. Its potential anti-aging properties are due to its power to alter the levels of proteins associated with senescence, such as adenosine 5′-monophosphate-activated protein kinase (AMPK) and sirtuins, and preventing pro-aging proteins, such as nuclear factor-kappa-B (NF-κB) and mammalian target of rapamycin (mTOR) [41]. Numerous studies have demonstrated mechanisms by which curcumin can act on the aging process; one of them investigated the molecular mechanism of curcumin in extending the lifespan of *C. elegans*, and the results of the study indicated that this action potentially occurred through increased resistance to OS and negative regulation of the AMPK signaling pathway [42].

Another study demonstrated how long-time curcumin therapy can progressively reverse cognitive dysfunction in D-gal-induced senescent mice, delaying the aging process, improving locomotor activity and cognitive functions, and restoring mitochondrial enzyme complex function [43]. It was further reported that curcumin supplementation rejuvenates senescence-related changes in the thymus among senescent mice caused by D-gal through promoting proliferating cells, protecting cells from apoptosis, and elevating transcription of the autoimmune regulator [44,45]

Despite the growing body of research indicating the potential benefits of curcumin in addressing age-related disorders, there remains a gap in the understanding of its precise mechanisms of action and optimal dosages for different age-related conditions. While some studies have shown promising results in terms of curcumin’s antioxidant, anti-inflammatory, and neuroprotective properties, there is still a need for more robust clinical trials and mechanistic studies to elucidate the specific pathways through which curcumin exerts its effects on aging-related processes. Additionally, variations in bioavailability and metabolism of curcumin among individuals pose a challenge in determining the most effective dosage and formulation for therapeutic purposes. Furthermore, the lack of standardized protocols and inconsistent reporting of outcomes in existing studies hinder the ability to draw definitive conclusions regarding the efficacy of curcumin in preventing or treating age-related disorders. Addressing these gaps through well-designed clinical trials, mechanistic investigations, and standardized protocols could provide valuable insights into the full potential of curcumin as a therapeutic agent for promoting healthy aging and combating age-related conditions.

The objectives of this systematic review are to evaluate the current scientific evidence on the effects of curcumin in preventing and managing age-related disorders, to assess the safety and tolerability of curcumin supplementation in older adults, to explore the mechanisms through which curcumin may exert its beneficial effects on aging-related processes, and to identify potential gaps in the literature that warrant further research. By synthesizing data from clinical trials and studies focusing on OS, inflammation, cognition, NDs, sarcopenia, and other age-related conditions, this review aims to provide a comprehensive overview of the therapeutic potential of *Curcuma longa* and curcumin in promoting healthy aging and improving quality of life in the elderly population.

## 2. Materials and Methods

### 2.1. Focal Question

This systematic review was carried out with the aim of answering the following question: “Can curcumin produce beneficial effects on conditions related to the aging process?”.

### 2.2. Language

Only clinical studies published in English were used.

### 2.3. Literature Search

This systematic review included clinical trials published in MEDLINE–PubMed, COCHRANE and EMBASE. The keywords used were *Curcuma longa* or saffron or turmeric or curcumin or curcuminoids and OS or inflammation or mitochondrial dysfunction or sarcopenia or cognition or memory or dementia or PD or AD or aging or NDs. These descriptors guided the identification of studies related to *Curcuma longa* and its effects on OS and inflammatory processes related to aging. S.M.B. and Y.C.N. carried out the identification and inclusion of studies. In the case of conflicting research results, a third judge ruled (M.J.S.M.).

### 2.4. Inclusion and Exclusion Criteria

The inclusion criteria for the studies were intervention studies in humans. Exclusion criteria were studies not published in English, editorials, conferences, letters to editors, reviews, poster presentations, and case reports.

### 2.5. Data Extraction

There was no time limit for the search for clinical trials in this review. For data extraction, we used the PICO format (Population, Intervention, Comparison, and Outcomes).

### 2.6. Study Selection

**Figure 2:**
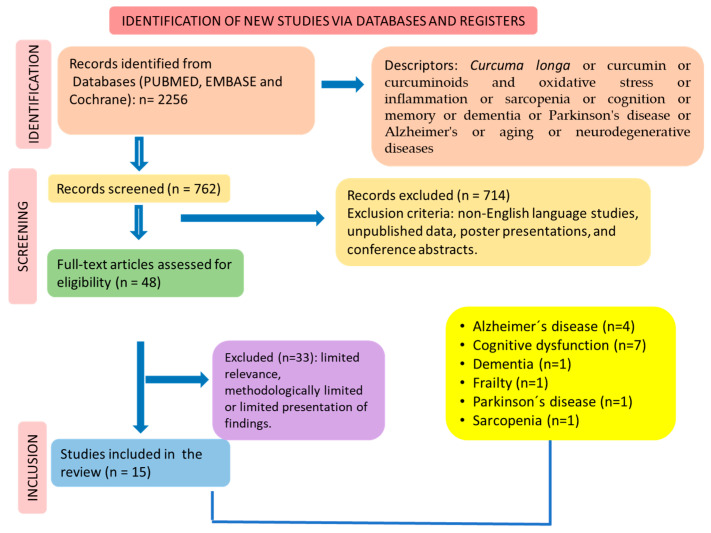
Flow diagram showing the study selection (according to PRISMA guidelines) [47].

The search/selection of studies was performed according to the guidelines of PRISMA (The Preferred Reporting Items for a Systematic Review and Meta-Analysis) [46,47] (Figure 2).

### 2.7. Quality Assessment

The Cochrane Handbook was used to assess the risk of bias related to the studies selected for systematic reviews of interventions [48].

### 2.8. Registration

This study was registered by PROSPERO under the ID CR42024559316.

## 3. Results

Initially, 2256 documents were identified according to the search terms. After applying the inclusion/exclusion criteria, we identified 15 clinical trials that met these criteria for synthesis. Figure 2 shows the selection of the studies in accordance with PRISMA guidelines.

## 4. Discussion

This review explores the beneficial effects of curcumin in addressing aging-related disorders, highlighting the intricate interplay between physiological and metabolic cascades that contribute to age-related impairments. Highlighting the critical roles that inflammation, OS, and mitochondrial dysfunction play in the pathophysiology of ailments like memory loss and cognitive impairment, NDs, frailty, and sarcopenia, the review highlights curcumin’s potential as a versatile therapeutic agent. By elucidating how curcumin’s antioxidant and anti-inflammatory properties may counteract these detrimental processes, the discussion sheds light on the mechanisms through which curcumin could mitigate age-related conditions. Furthermore, the review explores the impact of curcumin on neuronal growth factors, neuroplasticity, muscle protein synthesis, and degradation, offering insights into its potential to enhance brain functions and muscle health in the aging population.

### 4.1. Beneficial Effects of Curcumin and Aging-Related Disorders

**Figure 3:**
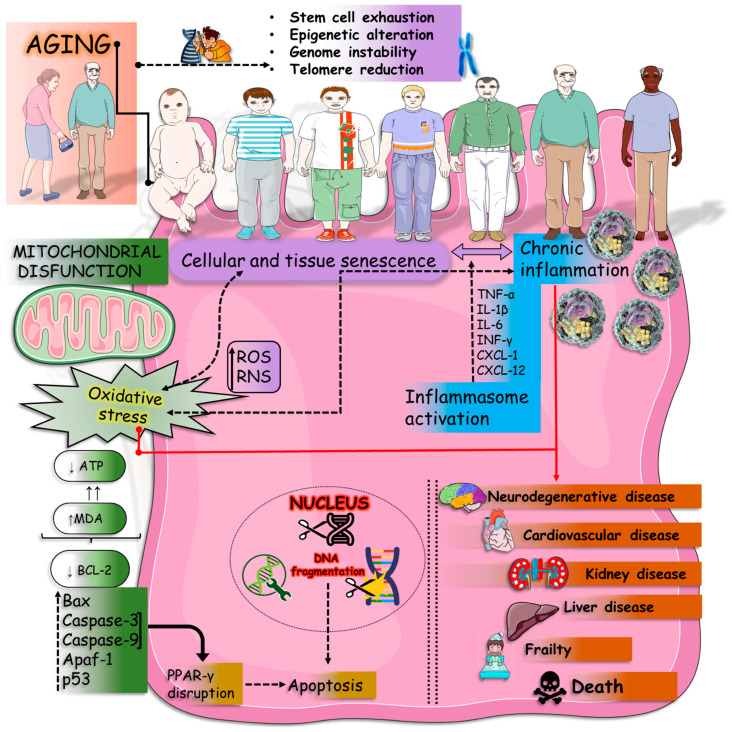
Overview of the aging process. Cellular senescence triggers inflammatory processes and the release of pro-inflammatory biomarkers such as IL-1β, IL-6, TNF-α, IFn-γ, CXCL-1, and CXCL-12. Free radicals and reactive oxygen species induce the installation of oxidative stress (OS) that aggravates inflammation. On the other hand, inflammation aggravates OS, leading to a vicious cycle. Apaf-1: apoptotic protease-activating factor-1; Bax: Bcl-2-like protein X; BCL-2: B-cell lymphoma 2; CXCL: chemokine (C-X-C motif) ligand; IL: interleukin; INF-γ: interferon-γ; MDA: malonaldehyde; TNF-α: tumor necrosis factor-α. ↓: decrease; ↑: increase.

Aging-related disorders are related to impairment in several physiological and metabolic cascades (Figure 3). These processes are profoundly linked to inflammation, OS and mitochondrial dysfunction, leading to several disorders such as impaired memory and cognition, NDs (such as AD and PD), frailty, and sarcopenia.

### 4.2. Inflammation

Inflammatory processes are crucial in the development of disease conditions related to the aging process. Curcumin’s anti-inflammatory activity involves regulating inflammatory signaling pathways and inhibiting the production of inflammatory mediators. Curcumin can bind to Toll-like receptors (TLRs) and downregulate mitogen-activated protein kinases (MAPKs), activator protein 1 (AP-1), and NF-κB signaling pathways that play an important role in inflammatory mediator generation. Moreover, curcumin, by inhibiting the NF-κB pathway, can directly restrain the assembly or even inhibit the activation of the NOD-like receptor pyrin domain-containing 3 (NLRP3) inflammasome, a cytosolic multiprotein complex involved in the development of several inflammatory diseases [36,49,50,51,52,53,54,55]. In addition, curcumin reduces inflammation through its antioxidant properties by inhibiting nicotinamide adenine dinucleotide phosphate (NADPH) oxidase and elevating the activity of antioxidant enzymes and consequently lowering reactive oxygen species (ROS) [49,56].

Another anti-inflammatory mechanism of curcumin is the nuclear factor erythroid 2–related factor 2 (Nrf2)-Kelch-like ECH-associated protein 1 (Keap1) pathway, which is strongly related to OS and inflammation by its association with NF-κB, MAPK, phosphoinositide 3-kinase (PI3K) and protein kinase C (PKC) pathways and by its role in regulating gene expression of antioxidant and detoxifying enzymes [57,58,59,60]. Furthermore, curcumin can block both the production of tumor necrosis factor (TNF)-alpha (TNF-α) and the cell signaling pathways mediated by TNF in various types of cells, decrease the release of several ILs through the downstream regulation of NF-κB by acting on peroxisome proliferator-activated receptor gamma (PPAR-γ), act as a natural free radical scavenger due to its chemical structure, and suppress pro-inflammatory pathways related to most chronic diseases [49,58].

Curcumin can also regulate immune cells such as dendritic cells, T regulatory cells, and T helper 17 (Th17) cells, which produce interleukin (IL)-17, IL-22, and IL-23, inducing an inflammatory response. Curcumin mainly inhibits Th17 differentiation and regulates Treg, which are anti-inflammatory cells, and induces Th17 balance by inhibiting the IL-23/Th17 pathway, maintaining immune homeostasis [35,36,49,61,62]. Also, it can induce the polarization of macrophages into an anti-inflammatory M2 phenotype. Furthermore, levels of pro-inflammatory mediators such as IL-1, IL-1β, IL-6, IL-8, IL-17, IL-27, nitric oxide (NO), inducible nitric oxide synthase (iNOS), C-X-C motif chemokine ligand 8 (CXCL8), C-C motif chemokine ligand 2 (CCL2), cyclooxygenase 2 (COX-2), granulocyte colony-stimulating factor (G-CSF), and monocyte chemotactic protein-1 (MCP-1) can be decreased by curcumin [20,32,49,63].

Curcumin can also work as an anti-inflammatory mediator by modulating the Janus kinase/signal transducer and activator of transcription (JAK/STAT) signaling pathways [64,65] and reducing macrophage infiltration and mRNA levels of macrophage M1 [66]. Moreover, the protective role of curcumin on gastric mucosal inflammation in mice induced by cisplatin occurred by decreasing IL-1β, IL-17, IL-23, TNF-α, and myeloperoxidase, enhancing levels of IL-10, and downregulating activation of NF-κB [67].

**Figure 4:**
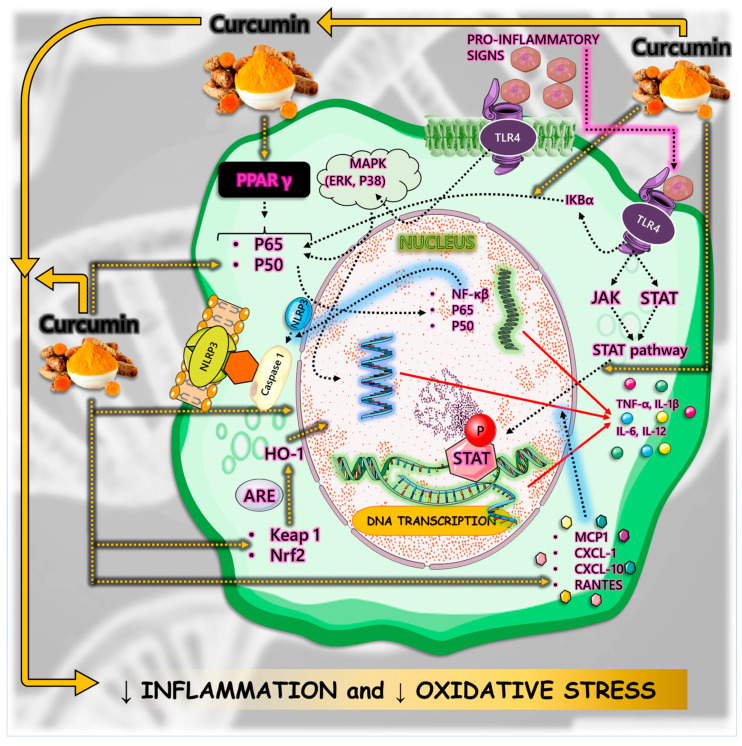
The effects of curcumin against inflammatory pathways. Curcumin inhibits the MAPK, ERK, p38, p65, p50, and NFkB pathways and the consequent release of pro-inflammatory cytokines such as interleukin (IL)-1; IL-12, and tumor necrosis factor-α (TNF-α). Besides that, curcumin also inhibits the Janus kinase/signal transducer and active factor of transcription (JAK-STAT) cascade. There is stimulation of Kelch-like ECH-associated protein 1 (Keap1) and nuclear factor erythroid-2 related factor 2 (Nrf2) that also interfere with pro-inflammatory pathways. ARE: antioxidant responsive elements;CXCL: chemokine (C-X-C motif) ligand; ERK: protein kinase RNA-like endoplasmic reticulum kinase; HO-1: Heme-oxygenase-1; IKB: IkappaB kinase; MAPK: mitogen-activated protein kinase; MCP1: monocyte chemoattractant protein-1; NF-κβ: nuclear factor-kappa beta,; NRLP34: NLR family pyrin domain containing; PPAR: peroxisome proliferator-activated receptor; RANTES: IL-8 superfamily cytokines; TLR4: Toll-like receptor. ↓: decrease.

Another point to bring to light is that inflammation is closely related to OS. The significant accumulation of free radicals such as ROS or reactive nitrogen species (RNS) leads to OS, which aggravates inflammation by stimulating the transcription of factors related to inflammation. Curcumin can decrease ROS production through NADPH oxidase and augment antioxidant molecules (such as catalase, superoxide dismutase, and glutathione peroxidase enzymes) activities, and is associated with Nrf2-Keap1 pathways [68,69]. Figure 4 summarizes the anti-inflammatory pathways modulated by curcumin.

Among the “pillars” of aging, inflammaging should be mentioned. This term is applied to define the close relationship observed between low-grade chronic inflammation and the aging process with no infectious conditions. This scenario is related to several impairments, including brain conditions. As already pointed out, the immune system has a critical role in the aging process or the “biological age”, which can be evaluated by metabolomics and genomics. Biological age is different from chronological age. Biological age may be influenced by genetics, lifestyle, and the environment. However, the immune system can be impaired substantially in the normal aging process, leading to great consequences for the body [70]. Curcumin can inhibit the formation of free radicals and other pro-inflammatory biomarkers related to age-related diseases [71,72].

### 4.3. Oxidative Stress

Aging is a gradual combination of important tissue and cellular changes, integrating structural, functional, and physiological changes, leading to functional disorders and increasing susceptibility to death. Named “hallmarks of aging”, this process is linked to molecular events such as dysregulated nutrient sensing, telomere wear, genomic instability, epigenetic changes, cellular senescence, loss of proteostasis, altered intercellular communication, and stem cell exhaustion [73,74,75,76,77,78,79,80]. OS and inflammatory processes are associated with the aging process. Various circumstances, such as stress, infections, exposure to inflammation, smoke, and radiation, produce ROS due to metabolism [81]. These molecules can lead to irreversible cellular damage when the endogenous antioxidant system or the intake of exogenous antioxidants is insufficient [82,83,84]. OS is linked to the genesis of numerous health conditions, both in the aging phase and even in the younger stages of life. These include obesity, hypertension, diabetes, cardiovascular diseases (CVDs), NDs, cataracts, and cancer [85,86,87,88].

Numerous antioxidants can contribute to preventing the effects of the aging process. Produced by exogenous and endogenous pathways, ROS can be attenuated by enzymatic and non-enzymatic antioxidants. There are several defense systems, including peroxidase, glutathione, catalase, superoxide dismutase, thioredoxin, cytochrome c oxidase (complex IV), representing endogenous antioxidants and vitamin E, coenzyme Q, carotenoids and ascorbic acid, representing some possibilities of exogenous antioxidants [89,90,91].

Cells constantly strive to maintain the level of ROS essential for their normal functioning. However, excessive production of ROS reduces the activity of the antioxidant enzymatic defense system and the content of non-enzymatic proteins (GSH), which compromises the general defense system and prevents it from eliminating excess free radicals [92,93]. ROS, produced in hyperoxia and inflammatory conditions, combined with a low and damaged antioxidant defense system, change the homeostasis of the biological system as a whole. In excess, they cause oxidative damage to deoxyribonucleic acid (DNA). They can react with them and attack nitrogenous bases and the sugar–phosphate skeleton, instigating single- and double-stranded DNA breaks, which are also linked to premature aging. Considering all these consequences, OS can stimulate various pathologies (chronic and acute), cause acute diseases (trauma and stroke), and accelerate aging processes [94,95].

As a dietary phenolic compound, curcumin is useful for longevity through declining OS, modulating signal transduction, and gene expression. Curcumin can extend shelf life by inhibiting lipid peroxidation and also increasing antioxidant activities [96]. It has enormous potential to minimize age-related cellular damage caused by the generation of ROS. It can stabilize Nrf2 and enhance the expression of heme oxygenase-1 (HO-1), in addition to stimulating the Nrf2 pathway, which is essential in the activation of antioxidant enzymes, such as thioredoxin reductase, heme oxygenase, sirtuins, and Hsp70 [97,98,99]. Curcumin can promote significant neuroprotective actions by modulating neuroinflammatory signaling pathways, scavenging ROS, and inhibiting or reducing the production of pro-inflammatory mediators [100].

An interesting study showed that, on sepsis-induced cardiac dysfunction, curcumin can activate sirtuin 1 (SIRT1), elevate the expression of mitochondrial biogenesis-related genes *Nrf2*, *Pgc1α*, and *Tfam*, reduce dynamin-related protein 1 transport to mitochondria, and restore mitochondrial morphology and function in heart cells [101].

Tetrahydro-curcumin, one of the most important metabolites of curcumin, was used in a study to remove ROS from hyperglycemia and increase the concentration of reduced glutathione (γ-glutamylcysteinyl glycine) in cultured rat lenses [102]. In another study, microsomal lipid peroxidation inhibition was reported in male rats’ liver supplemented with 1% turmeric [103]. Regarding the effects of curcumin on several target molecules that are directly or indirectly related to different metabolic functions [104], a study reported an increase in cellular antioxidant defenses in rats subjected to treatment with cyclophosphamide to stimulate lung injury, fed with curcumin for seven days before receiving the treatment [105]. Curcumin functions as a biochemical antioxidant; it can extend lifespan by inhibiting lipid peroxidation and increasing antioxidant activities, and as a dietary phenolic compound, it can promote longevity by reducing OS and modulating gene expression [106,107].

### 4.4. Mitochondrial Dysfunction and Apoptosis

Mitochondrial dysfunction is related to several aging conditions and disorders. Deregulated levels of ROS can potentially cause oxidative damage in the mitochondrial DNA (mtDNA), affecting the organelle’s function and inducing redox signaling to the other cell organelle [59,108]. Then, mitochondrial dysfunction is characterized by higher NO synthesis, OS, and lower ATP production and oxygen consumption. However, mitochondria have ROS scavenging systems where superoxide dismutase turns superoxide radical into hydrogen peroxide. It suffers the action of catalysts such as glutathione peroxidase, which breaks hydrogen peroxide into water.

Curcumin can improve antioxidant activity and reduce oxidative damage in mitochondria by increasing the effect of superoxide dismutase, glutathione, and catalase and also inhibiting ROS-generated enzymes such as cyclooxygenase, lipoxygenase, and xanthine hydrogenase/oxidase [109,110].

In mice with chronic kidney disease-induced mitochondrial dysfunction, curcumin could improve mitochondrial biogenesis and mitochondrial function and suppress OS, probably by inhibiting glycogen synthase kinase-3β (GSK-3β) activity. Curcumin modulated levels of mitochondrial ATP and the basal mitochondrial oxygen consumption rate; it also attenuated mitochondrial superoxide production. Furthermore, curcumin could inhibit the deleterious alterations in mitochondria morphology and enhance the expression levels of mitochondrial transcription factor A (TFAM), nuclear respiratory factor 1 (NRF-1), and PPAR-γ coactivator 1-α (PGC-1α), which were decreased in mouse muscle [111,112].

Furthermore, by improving mitochondrial function, curcumin pretreatment protected rat bone marrow mesenchymal stem cells against hypoxia and reoxygenation injury. The pretreatment improved ATP production and reduced ROS formation and changes in mitochondrial membrane potential, which are caused by the excessive levels of ROS that alter mitochondrial membrane permeability and can lead to apoptosis. In addition, curcumin also prevented hypoxia and reoxygenation-induced cell viability reduction and improved nuclei morphology [113].

Curcumin has been shown effective in inducing apoptosis in different cancer cells, mainly due to its potential to inhibit the phosphatidylinositol 3-kinase/protein kinase B (PI3K/AKT) pathway, whose activation hinders apoptosis by upregulating anti-apoptotic genes such as B-cell lymphoma 2 (Bcl-2) and downregulating pro-apoptotic genes like Bcl-2 associated protein X (Bax) [114,115]. The PI3K/AKT pathway inhibition by curcumin can be explained by the upregulation of phosphatase and tensin homolog (PTEN). This tumor suppressor downregulates PI3K/AKT signaling and gene expression in AKT activation [115]. Also, curcumin inhibits the tyrosine kinase epidermal growth factor receptor (EGFR), which activates the PI3K/AKT pathway and induces apoptosis in neoplastic cells [116].

The JAK/STAT pathway is also a signaling pathway that regulates apoptosis and is affected by curcumin in a myeloproliferative neoplasm model. In this experiment, curcumin inhibited JAK2/STAT and mammalian target of rapamycin complex 1 (mTORC1) pathways in JAK2 V617F-mutated cells, inducing apoptosis [117]. Furthermore, curcumin presented the ability to sensitize neoplastic cells to suffer apoptosis mediated by death receptor pathways, which are unlocked with death ligands, like TNF and Fas ligand, culminating in caspase-8 activation and consequent apoptosis. Notwithstanding, curcumin can induce endoplasmic reticulum stress-induced apoptosis pathway in tumor cells [115].

**Figure 5:**
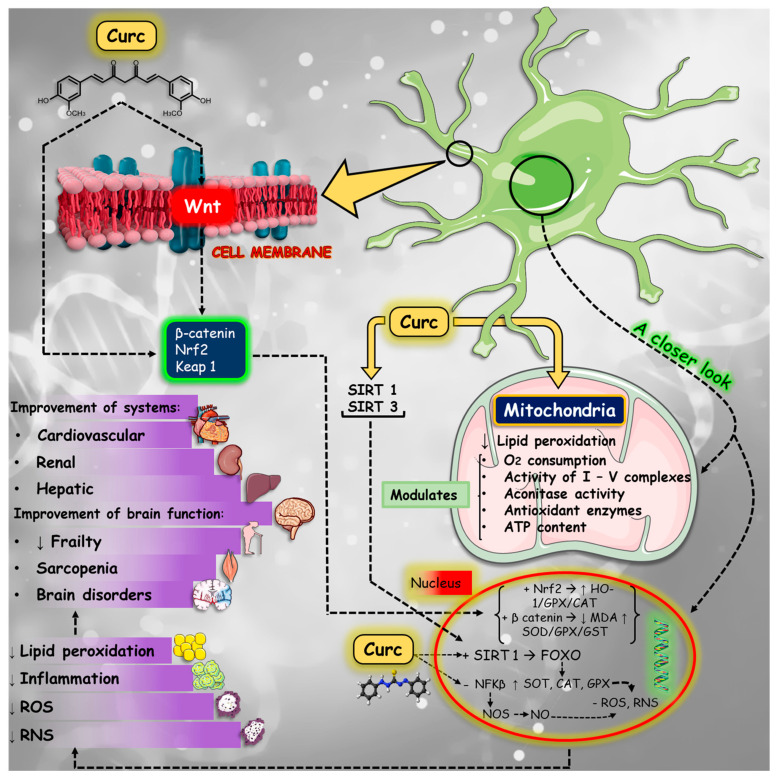
Effects of curcumin on mitochondrial dysfunction and oxidative stress in different signaling pathways. Curcumin can upregulate sirtuin (SIRT), Ketch-like ECH-associated protein 1, nuclear factor erythroid-2 related factor 2 (Keap1-Nrf2), and Wnt/β catenin pathways, and inhibit nuclear factor kappa beta (NF-κβ). The results of the stimulation and inhibition of these pathways is the modulation of lipid peroxidation, oxygen consumption, aconitase and antioxidant enzyme modulation, and ATP production in mitochondria. Moreover, curcumin is related to the upregulation of the synthesis of glutathione peroxidase (GPX), superoxide dismutase (SOD), reduction of malonaldehyde (MDA), reactive oxygen species (ROS), and reactive nitrogen species (RNS). The results of curcumin effects are improvements in cardiovascular, renal, and hepatic diseases. Furthermore, there is a reduction in frailty, sarcopenia, and brain disorders. ↓: decrease; ↑: increase.

Curcumin also showed inhibitory effects on apoptosis in non-cancerous diseases, which enhances its therapeutic applications. In diabetic cardiomyopathy, it reduced ROS and activated PI3K-AKT signaling pathways, resulting in the downregulation of Bax and caspase-3 expression and consequent apoptosis inhibition [118]. In a septic acute kidney injury mouse model, curcumin showed anti-inflammatory capacity and attenuation of apoptosis by JAK2/STAT3 and NF-κB signaling pathway inhibition, resulting in increased levels of Bcl-2 and decreased levels of Bax and caspase-3 [119]. Figure 5 summarizes some curcumin effects related to mitochondrial function and reduction of OS.

### 4.5. Neurodegenerative Diseases

NDs include a heterogeneous group of neurologic conditions that covers dementia predominantly, multiple sclerosis, amyotrophy lateral sclerosis, AD, and PD, and can lead to neural cell death [120,121,122,123,124].

Usually, NDs are irreversible, progressive, and related to loss of function; as the structures degenerate, a gradual and progressive loss of motor skills and/or cognitive skills can lead to loss of function, debilitation, and mental impairment. Neuroinflammation and OS are pathophysiologies common to all forms of ND. Among the most common NDs are AD (the most frequent), PD, and amyotrophic lateral sclerosis; in AD, the symptoms include progressive and irreversible cognitive deficits that may present with changes in behavior and mood, and memory loss is also common as the disease progresses; it corresponds to 60% to 80% of dementia cases, and more than 30 million people have this condition. These diseases affect most elderly individuals but can also occur at other ages and are characterized by having a constant progressive course due to the increasing decrease in specific neurons in the brain [125,126,127,128,129,130,131,132].

Therapeutic resources that only treat the symptoms and prevent the progression of the disease are used at the moment, such as drugs including cholinesterase inhibitors used for AD that do not change the course of the disease and only provide improvement in symptoms of behavioral deficits. Still, several patients exhibit little effectiveness in the therapeutic response due to the difficulty of adherence to treatment that has a high cost, physiological variability, and adverse effects, which include nausea, vomiting, dizziness, and diarrhea [133,134,135,136].

**Figure 6:**
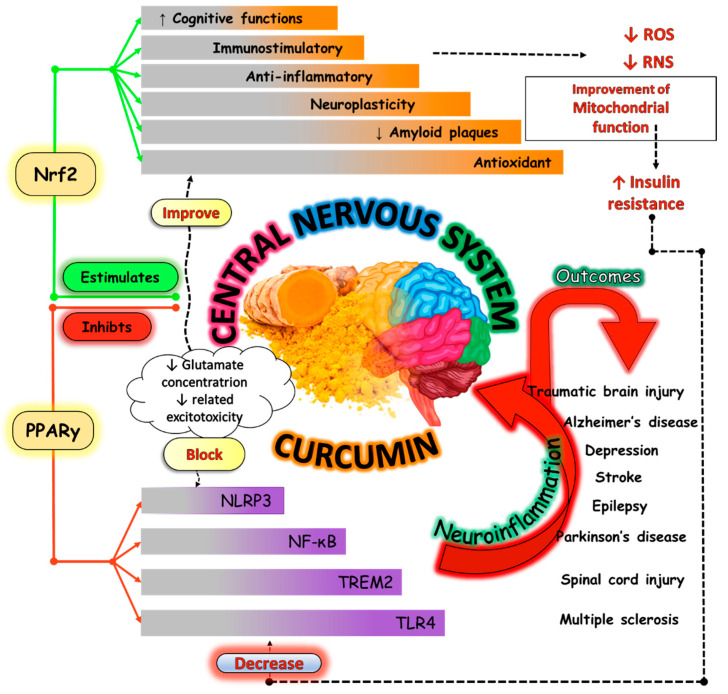
Curcumin and its effects on nervous system disorders. Activation of NOD-like receptor pyrin domain-containing 3 (NLRP3), Toll-like receptor 4 (TLR4), nuclear factor-kappa beta (NF-κβ), and triggering receptor expressed on myeloid cell 2 (TREM2) is associated with neuroinflammation and the risk of developing conditions such as AD and PD, brain injury, depression, and multiple sclerosis. However, curcumin can block PPAR-γ, which is an important mediator for the expression of these inflammatory factors. In addition, curcumin can stimulate nuclear factor erythroid-2 related factor 2 (Nrf2) and leads to improvement of inflammation, OS, cognitive functions, neuroplasticity, and memory. This activity can result in a decrease in ROS and RNS, improving mitochondrial function, and a decrease in insulin resistance, which reduces the activity of the inflammatory factors mentioned. Furthermore, it can reduce β amyloid plaque accumulation to avoid future inflammation of the nervous system. ↓: decrease; ↑: increase.

These reasons led to the need for other therapeutic approaches, and *Curcuma longa*, due to its antioxidant, immunomodulatory, and anti-inflammatory properties, may be an option. Mechanisms such as glucose metabolism and endothelial function are closely linked to processes of neurogenesis, neuroinflammation, and synaptic plasticity, and improvement of the function and structure of synapses, regulation of proteins, and delay of the neural dysfunction process has been associated with the consumption of curcumin [137,138,139]. Figure 6 shows some effects of curcumin on the prevention of ND.

#### 4.5.1. Cognition

Although crystallized intelligence remains unchanged, neural aging leads to important changes in fluid intelligence as age advances, resulting in impairments in attention, memory, processing speed, and visuospatial and psychomotor abilities [140,141]. The pharmacological class most used in these cases is acetylcholinesterase (AChE) enzyme inhibitors, as therapeutic options are limited. However, numerous studies have shown that curcumin has relevant effects on cognitive function [142,143].

Zhi et al. [44] investigated male C57BL/6 mice with trigeminal neuralgia administered 100 mg/kg/day curcumin twice a day for 14 days to observe its effects on orofacial allodynia and cognitive impairment and showed an increase in the density of dendritic spines, and in the regulation and proportion of dendritic spines, relieving the synaptic damage neurons in the hippocampus, in addition to increased mechanical and cold pain thresholds and improved spatial learning and memory deficits. As also presented in male Otsuka Long-Evans Tokushima Fatty rats as a model of spontaneous DM2, they were subjected to physical exercise or physical exercise in combination with 5 g/kg of curcumin to investigate their cognitive responses; then, it was evidenced that both groups showed a significantly lower escape latency and a longer swimming time spent in the target quadrant; however, the best results were seen in the group in combination with curcumin, indicating a decrease in learning and memory deficits [144].

The improvement in cognitive function may be partially linked to an increase in neurogenesis, as analyzed in male Sprague Dawley rats in a model of Gulf War illness treated with 30 mg/kg curcumin for 30 days and exposed to the object localization test to evaluate cognitive function, as well as the new object test to analyze recognition memory and measurement of hippocampal neurogenesis. The results observed were a greater exploration time of both an object moved to a different location and a new object over a familiar object, as well as an increase in hippocampal neurogenesis; in addition, the improvement in cognitive function may be partially linked to greater neurogenesis [145].

Behind all cognitive impairment, there is an exacerbated OS mechanism due to mitochondrial changes caused by aging, which trigger a theory known as “free radical aging”. It is explained by the reduction in ATP production, culminating in lower consumption of oxygen, followed by a higher concentration of free molecules (O_2_) to bind with NO, generating peroxynitrite (ONOO^−^) or hydroxyl radicals (^•^OH). This exacerbated production of ROS unbalances antioxidant activities mediated by the enzymatic action of superoxide dismutase and glutathione peroxidase, increasing lipid peroxidation and the oxidation of DNA and proteins, thereby, in addition to stimulating the release of pro-inflammatory and pro-apoptotic factors, causing apoptosis and autophagy of neural cells [146,147].

Using 100 mg/kg of curcumin one hour before treatment with cisplatin (an important chemoattractant that can lead to cognitive impairment) in C57BL/6 mice showed that the autophagy caused by cisplatin was induced through the transcription factor 4/protein kinase B/the mammalian target of the rapamycin (ATF4-Akt-mTOR) signaling pathway by endoplasmic reticulum stress. The analysis also showed that curcumin increased the activation of the AMPK/c-Jun N-terminal kinase (JNK), culminating in the inhibition of Akt and mTOR and upregulation of the Bcl-2 protein (anti-apoptotic), suppressing apoptosis, being associated with increased neurogenesis and synaptogenesis in the hippocampus, and clarifying the improvement and recovery of cognitive function, as seen in the MWM test (improved spatial learning and memory) and NORT (increased recognition function). In addition, it increased the amount of cells positive for the marker of neurogenesis doublecortin in the hippocampus and the density of the dendritic spine, and inhibited the levels of Bax- and Bcl-2-interacting mediator of cell death (Bim) proteins (pro-apoptotic members); the opposite is seen when cisplatin is administered alone, which despite presenting a considerable level of autophagy also induces an exaggerated increase in apoptosis, attenuating neurogenesis and synaptogenesis, explaining cognitive impairment [148].

Rueda et al. investigated the actions of 300 mg/kg of curcumin in prenatal or early postnatal stages on geomorphology and cognition in pregnant female mice with Ts65Dn Down syndrome [149]. The use of curcumin acted to increase brain weight, the density of positive bromodeoxyuridine and 4’6-diamidino-2-phenylindole in the hippocampus of those on short-term prenatal and short-term postnatal treatment, and the levels of postsynaptic density protein 95 and synaptophysin. There was a decrease in escape latency, and in the probe test, there was an increase in the number of crossings in the platform position, indicating an improvement in cognitive status and neuromorphology.

#### 4.5.2. Memory

As noted above, using curcumin as a drug therapy adjuvant shows several neurological benefits, including preserving and improving memory and learning, as seen in research using rodents with neurological deficits [150,151,152,153,154,155].

The study by Changleck et al. investigated the effects of curcumin on lead-induced inflammation and cholinergic dysfunction in male ICR mice [156]. It was possible to demonstrate that the groups receiving treatment associated with curcumin showed a significant increase in AChE levels in the brain, decreased TNF-α, COX-2, phosphorylation of inhibitory kappa B kinase beta (IKKβ), extracellular signal-regulated kinase (ERK), and JNK. Moreover, an improvement in spatial memory was observed, especially in those undergoing treatment with curcumin 200 mg/kg. Another study analyzed the effects of 50 mg/kg/day curcumin supplementation for 4 weeks on memory deficits, lactate content, and monocarboxylate transporter 2 (MCT2) in a model of sixteen amyloid precursor protein (APP)/presenilin 1 (PS1) transgenic male and female mice [157]. The analyses demonstrated that the group treated with curcumin showed an increase in escape length and platform passage time, indicating an improvement in memory deficit, as well as lactate levels and protein MCT2, which were significantly increased in the cerebral cortex and hippocampus.

The effects of consuming 5, 15, and 45 mg/kg of curcumin associated with cholinergic drugs on the cholinergic system of male Wistar mice, compared to the isolated administration of agonists (nicotine, pilocarpine) and cholinergic antagonists (succinylcholine and scopolamine), demonstrated a significant improvement in memory retention in those undergoing treatment with agonists [158].

Ikram et al. investigated the neuroprotective mechanisms of dietary supplementation of 50 mg/kg of curcumin for 6 weeks on male C57BL/6N mice and HT22 cells from the hippocampus of mice with neurodegeneration [159]. The authors observed that ethanol increased ROS, lipoperoxidation, Toll-like receptor-4 (TLR4) expression, and receptors for advanced glycation end products (RAGEs); concomitantly, there was also an increase in phosphorylated (p)-JNK, p-NF-κB, apoptotic markers (Bax, cleaved caspase-3, and PARP-1), and decreased anti-apoptotic markers; these were responsible for neuroinflammation, neurodegeneration, and synaptic dysfunction. When the curcumin was administered, there was both in vitro and in vivo a decrease in ROS and an increase in the expression of Nrf2/HO-1, an important cytoprotective and detoxifying agent. Furthermore, chronic use of curcumin was associated with an inhibition of apoptotic markers and an increase in Bcl-2, indicating a rescue against neurodegeneration and memory impairment that was confirmed through Nissl staining and FJB of neuronal cells.

Zhang et al. demonstrated that the acute use (single dose) of 50, 100, and 200 mg of curcumin showed no benefit on the spatial memory of male Sprague Dawley rats with memory deficit induced by a ventricular injection of β-amyloid peptide (1–42); the opposite was seen in those on chronic treatment with the same dosages of curcumin, with a significant decrease being observed in escape latency, and an increase in the frequency of crossing the platform location and in spatial preference for the target quadrant, especially when using doses of 100 mg or 200 mg of curcumin [160].

#### 4.5.3. Alzheimer’s Disease

AD is marked mainly by accumulations of extracellular amyloid plaques, composed of β-amyloid peptides (Aβ) responsible for directly inducing tau hyperphosphorylation and neurite degeneration and intracellular neurofibrillary tangles, composed by hyperphosphorylated microtubule-associated protein (MAPT) tau protein, which disrupts microtubules and impairs axonal transport in the brain [161,162,163]

Besides that, both aging and the accumulation of Aβ deposits pathologically stimulate microglia and astrocytes, inducing inflammatory processes with the intense release of pro-inflammatory biomarkers, such as IL-8, TNF-α, IL-1β, and IL-γ, with an increase in the enzymatic activity of beta-secretase and gamma-secretase due to decreased action of the AMPK pathway, which is dependent on JNK, cleaving the beta-APP and leading to the formation of more Aβ aggregates that bind to the RAGE, increasing the concentrations of ROS and RNS, stimulating the activation of NF-κB, which will trigger the production of NO. OS leads to a decrease in the activity of antioxidants, including glutathione, catalase, and glutathione-S-transferase, with a consequent increase in lipid peroxidation and nitrite levels. This inflammatory cascade is responsible for neuroinflammation and neurodegeneration [164,165,166,167,168,169].

Fortunately, in addition to AChE inhibitor medications, studies have shown that curcumin therapy acts protectively in the pathogenesis of AD, reducing OS and inflammation, in addition to inhibiting the formation of Aβ fibrils from Aβ40 (1–40) and Aβ42(1–42) and amyloid plaques [170,171,172,173,174,175].

The blood–brain barrier, which prevents most drugs from reaching their targets, makes the central nervous system the final frontier in drug delivery [176,177]. Yang et al. showed that in oral ingestion or peripheral injection of curcumin in Swedish Mutant (APPsw) Tg2576 transgenic mice, there was a blood–brain barrier crossing of curcumin that binds to amyloid plaques in vivo and in vitro [178]. With increasing doses of curcumin, it was possible to see a significant inhibition of Aβ aggregation and an induction of disaggregation of pre-aggregated Aβ40 in vivo. Furthermore, curcumin’s aggregation-inhibiting effects were superior to those demonstrated by non-steroidal anti-inflammatory drugs such as naproxen and ibuprofen. A second analysis carried out in APPsw AD Tg2576 transgenic mice treated with a low dose of curcumin (160 ppm) or a high dose (5000 ppm) showed declines in the levels of IL-1β and oxidized proteins, a 16.5% decrease in an astrocyte marker—glial fibrillary acidic protein (generally elevated in inflammatory conditions)—and 39.2% × 43% of insoluble and soluble Aβ levels in the entorhinal cortex and hippocampus. To conclude, a reduction in amyloid load by 43.6% and 32.6% in the average number of plaques was observed [171].

In a mouse model of AD, supplementation with 200 mg/kg of curcumin promoted a decrease in neurofibrillary degeneration and loss of hippocampal neurons, as well as a decline in Bax levels and an increase in Bcl-2 rates (decrease in apoptosis with a blockade of cytochrome c release from mitochondria) [179].

Under curcumin treatment, Kunming mice with irreversible brain lesions presented an increase in superoxide dismutase levels and a decline in brain malonaldehyde (MDA), as well as upregulation of Nrf2, NAD(P)H quinine oxidoreductase 1 (NQO1), HO-1, and γ-glutamyl cysteine synthetase (γ-GCS) in brain cells. These actions led to a significant improvement in OS, and a positive evolution in memory and spatial learning [180]. Similar results were shown in a study on synaptosomes obtained from the cerebral cortex of rats with neurodegenerative damage induced by Aβ1-42 in combined treatment with boric acid and curcumin. There were significant reductions in MDA and AChE and an increase in synaptophysin [181].

Aβ aggregation and tau hyperphosphorylation can also be induced by the interaction between p25 with glycogen synthase kinase 3 (GSK3β), and cyclin-dependent kinase 5 (Cdk5) [182,183]. A mouse model of AD induced by scopolamine or Aβ1-42 treated with curcumin showed a significant decline in lipid peroxidation and increased superoxide dismutase levels, in addition to a reduction in Aβ aggregation and tau hyperphosphorylation through the regulation of GSK3β, Cdk5, p35, and p25 [184,185].

Curcumin also has an inhibitory role on the thioredoxin-interacting protein (TXNIP)/NLRP3 inflammasome pathway and the JNK-NF-κB signaling cascade through the controlled regulation of AMPK, causing a decrease in neuronal apoptosis due to lower levels of pro-caspase 1 and consequently lower levels of IL-1β and IL-18 [186,187], since the activation of AMPK provides the suppression of inflammation and OS [188]. As analyzed in a group of male C57BL/6 J mice in an AD model, the use of 150 mg/kg of curcumin improved spatial learning and spatial working memory and led to a decrease in lesions and apoptosis of neural cells; in addition, the deposition of Aβ1–42 and neuroinflammation were significantly reduced, reducing the levels of TNF-α, IL-6, IL-1β, and MDA, and augmenting the levels of superoxide dismutase and activation of the AMPK pathway [189].

The use of curcumin nanomaterials has also been widely used in therapeutic environments by increasing the aqueous solubility and bioavailability of curcumin in target tissues [190,191]. Therefore, nanomaterial composed of curcumin led to improvement in spatial learning and memory retention. Besides that, it promoted neurogenesis through increasing brain-derived neurotrophic factor (BDNF) levels and improved neuroinflammation by inhibiting the NLRP3 inflammasome activation pathway induced by Aβ deposition, through decreasing IL-18, CD68, and NLRP3 both in the hippocampus and the cortical area. A second nanomaterial with curcumin also reduced/inhibited neuronal death, as NLRP3 promotes the synthesis of a speck-like protein associated with apoptosis containing a CARD (ASC), a protein that recruits and activates pro-caspase-1 and IL-18 contributing to the activation of neuronal death [192]. Another nano curcumin administered once a week for three months in AD Tg2576 transgenic mice also provided a lower density of amyloid plaques in the hippocampal region through thioflavin T (ThT) staining and an improvement in working memory and signaling, observed through contextual fear conditioning tests and RAM [193].

#### 4.5.4. Parkinson’s Disease

The second most common ND, PD, is also progressive and irreversible, and around 1% of individuals over 50 years old are affected [194,195,196,197]. The loss of dopaminergic fibers of the brain and the progressive worsening of motor symptoms characterizes the disease that, as it progresses, leads to the loss of 50 to 70% of all dopaminergic neurons of the patient in the substantia nigra [131,196,198,199].

There is an increased amount of evidence that implicates OS and immunological alterations in the pathogenesis of PD. The accumulation of excessive ROS and other free radicals overloads the decaying dopaminergic neurons and creates an environment conducive to this disease [200]. Possible contributors to OS have been associated with the process of PD such as mitochondria, endoplasmic reticulum, dopamine, and α-Sinuclein, and it seems that their interactions and not their actions collaborate for progressive neurodegeneration [201]. Neuroinflammation can be very important for the pathogenesis of PD since it has destructive repercussions on the nigrostriatal dopaminergic pathways, which activate brain glial cells (especially microglia and astrocytes) to release several soluble factors that may be neurotoxic and/or pro-inflammatory [199,202,203,204].

For its anti-inflammatory and antioxidant properties, curcumin has been widely used as a food additive and as an adjuvant therapy to prevent or treat ND [205,206]. One study showed that it is possible to reduce deficits related to PD by raising the level of antioxidant enzymes only with curcumin [207]. It can also protect black matter from cells, inhibit apoptotic signaling pathways with NF-κB, decrease lipid peroxidation and protein aggregates, and also OS damage to the mitochondrial membrane [125,208].

Curcumin can promote neuroprotection and inhibit the α-Synuclein aggregation in a PD model. Its use inhibited NFκB activity protein stimulated by lipopolysaccharide. It can reduce the production and aggregation of α-synuclein. The authors of this study suggested that curcumin can be an option as adjuvant therapy for the management of PD and other synucleopathies [209].

Other authors investigated the effects of iron oxide nanoparticles capped with curcumin (FeONPs-Cur) in motor imbalance and neurochemical modifications in a PD model (reserpine induction). These animals showed a significant reduction in motor activity associated with a decrease in 5-hydroxytryptamine, norepinephrine, and dopamine and increased levels of malonaldehyde, nitric oxide, and monoamine oxidase. Glutathione, Na+/K+/ATPase, and AchE significantly decreased in both brain areas. Using iron oxide nanoparticles capped with curcumin (FeONPs-Cur) restored homeostasis and prevented motor deficits, suggesting that FeONPs-Cur could be an antiparkinsonian candidate [210].

### 4.6. Fragility

Fragility is characterized by excessive vulnerability of the individual to endogenous and exogenous stressors, which in general leads to a high risk of developing negative situations related to health and disability, accentuating the prevalence of chronic diseases [74,211,212,213,214]. Besides the existence of several fragility evaluation instruments, there is still no agreement on a standard instrument identifying fragility [215]. In one of them, the methodology conceptualizes frailty as an energy imbalance syndrome that culminates in slowness, fatigue, decreased muscle mass, physical activity, and strength [216]. It detected older adults with a higher risk of falls, mortality, and adverse events after surgery [217,218,219,220]. Studies with geographic coverage and methods of selection of diverse samples have shown estimates of frailty prevalence among older people [221,222,223].

A study in the United States showed that 15% of the non-basilar elderly population is fragile and 45% pre-fragile. There is a prevalence of frailty among women, older people, and racial and ethnic minorities. Despite these characteristics, there was substantial variability in the prevalence of frailty among demographic regions. There is a marked increase in the prevalence of chronic diseases and disabilities with frailty [211,214,224].

Curcumin, at a dose of 2.5 g 2 times daily and 150 mg of lipid curcumin nanoparticles (Theracurmin) 2 times daily, may have beneficial effects on muscle recovery, reducing the expression of muscle damage moved by exercise, reducing the loss of maximum voluntary contraction and minimizing the increase in blood levels of creatine kinase [225,226] which shows that the use of curcumin can be an important adjuvant in the control of pain caused by loss of muscle mass [227].

A pilot study (phase IIb clinical trial) showed that the use of curcumin C3 Complex^®^ can improve muscle strength and physical function in older people (sedentary and >65 years with c-reactive protein > 1 mg/dL) at risk for mobility disability [228].

### 4.7. Sarcopenia

Sarcopenia is characterized by loss of muscle mass, functionality, and strength and is an important factor in loss of mobility and frailty in the elderly [229,230,231,232,233,234]. It is estimated that by 2050, there will be 426 million people aged 80 years or more and 2.1 billion people aged 60 years or more. Sarcopenia is linked to several comorbidities besides disability, such as osteoporosis, DM2, and obesity [235,236,237]. It is a multifactorial pathogenesis that encompasses various mechanisms such as insulin resistance, anabolic resistance, malnutrition with decreased availability of amino acids, and chronic inflammation [238,239,240]. In 2019, an estimate suggested that the economic expenses related to sarcopenia in the United States were USD 40.4 billion, an average of USD 260 per person [241].

One study demonstrated that low-grade persistent inflammation impacts muscle protein degradation and synthesis by several signaling pathways impacting sarcopenia; low-grade inflammation is a symptom of cells that begin the senescence phase and exit the cell cycle. During aging, pro-inflammatory TNF-α, C-reactive protein, and IL-6 are somewhat elevated in circulation [242]. It has also been reported that older people with sarcopenia show significantly higher levels of circulating IL-6 and TNF-α [243] and that high levels of IL-6 and CRP increase the risk of loss of muscle strength [244]. A longitudinal study of 10 years also demonstrated that plasma levels of TNF-α, IL-6, and IL-1 were reliable biomarkers of morbidity and mortality in elderly participants [245].

Due to the relevance of inflammation and OS in the origin of sarcopenia, compounds with antioxidant and anti-inflammatory effects have the potential to act as complementary to current treatments for this disease. Curcumin is one of the potential compounds with these characteristics. Curcumin has also been reported to target a class of signaling molecules that alter cellular functions and exert their therapeutic effects; it has been linked to numerous health benefits in several studies, including muscle health [246,247,248,249].

The preservation of muscle mass in the course of aging is paramount for the prevention of sarcopenia. Studies reported that curcumin increased muscle mass without altering body mass in F344XBN rats at 32 months of age, supplemented with a 0.2% diet for 4 months. These results were similar to those reported in previous studies [250,251,252,253]. Another study with 12-month-old male Sprague Dawley rats that had LPS-induced sarcopenia and received treatment with 150 mg/kg of curcumin for two months showed improvement in muscle endurance, pressure strength, and fat/lean ratio [254]. The supplementation of curcumin (40 and 80 mg/kg) 30 min before forced exercise in 10-month-old ICR rats for 28 days could complement exercise-based therapy to prevent muscle problems such as sarcopenia systematizing the expression of genes associated with protein synthesis, inflammation, and apoptosis in chronic forced exercise [255].

The protective effects of curcumin in muscle atrophy induced by dexamethasone using differentiated C2C12 cells were evaluated and showed that treatment with curcumin reduced the expression of Murf-1 and Atrogin-1, preventing protein degradation. It also increased the level of Akt phosphorylation, an essential protein in the mTOR signaling pathway that stimulates protein synthesis and prevents protein degradation [256]. The significant increase, impairment, and recruitment of satellite cells to delay the onset of pre-sarcopenia and sarcopenia were demonstrated in a study in 18-month-old C57BL6J and C57BL10ScSN mice, which received 120 µg/kg of curcumin for six months. The increase in the proportion of positive satellite cells for MyoD isolated from muscles of the aged posterior limbs and the development of sustained myofibers in the aged soleus muscle showed this [234,247].

**Figure 7:**
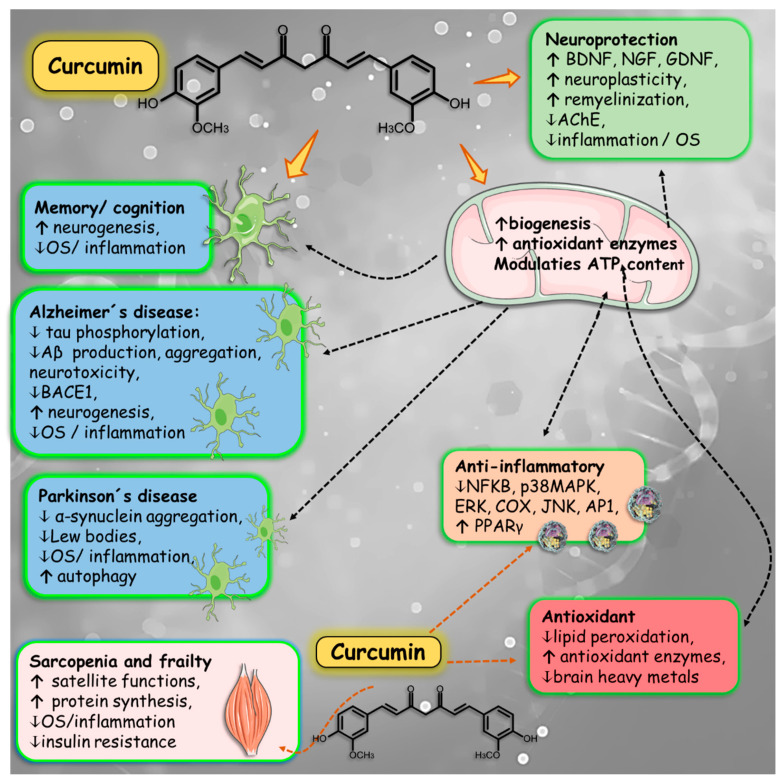
Summary of curcumin effects on some aging-related conditions. Curcumin possesses antioxidant and anti-inflammatory effects that are related to the prevention or treatment of memory loss, neurodegenerative diseases, sarcopenia, and frailty. These effects can play a role in mitochondrial functions that, on the other hand, are also associated with diminishing oxidative stress and inflammation. The results are associated with an increase in the synthesis of neuronal growth factors such as BDNF, NGF, and GDNF, an increase in neuroplasticity, reduction in brain neuroinflammation, and restoration of brain functions. In muscles, there is an increase in protein synthesis and a reduction in its degradation. AChE: acetylcholine esterase; AP-1: activator protein-1; BACE1: β-secretase 1; BDNF: brain-derived neurotrophic factor; COX: cyclooxygenase; ERK: extracellular signal-regulated kinase; GDNF: glial cell-derived neurotrophic factor; JNK: c-Jun N-terminal kinase; NGF: nerve growth factor; NF-κB nuclear factor kappa beta; p38MAPK: p38 mitogen-activated protein kinase; PPAR-γ: peroxisome proliferator-activated receptor gamma.

Evidence shows that curcumin is an alternative treatment with the potential for sarcopenia control; it can maintain the number and function of satellite cells, protect the mitochondrial function of muscle cells, and suppress OS and inflammation, thus achieving muscle protection [247]. Figure 7 is a summary of the effects promoted by curcumin in some aging-related conditions discussed above.

### 4.8. Depression

Depression is a chronic, recurrent, and frequent psychiatric disorder that profoundly influences quality of life and reduces the risk of death [257]. According to forecasts from the World Health Organization, it is estimated that it will become the main global burden of disease in the world by 2030 [258,259]. Significant personal suffering and economic loss result from the increased risk of suicide and worldwide morbidity resulting from depression [260,261,262,263,264]. In the elderly, it mainly affects those affected by chronic medical illnesses and cognitive impairment, causing suffering, disability, and family disruption, in addition to aggravating various diseases and increasing mortality. The processes associated with aging and diseases, such as arteriosclerosis and inflammatory, immunological, and endocrine changes, impair the integrity of the frontostriatal pathways, the hippocampus, and the amygdala, increasing susceptibility to depression [265,266].

Currently, there are several types of traditional antidepressant medications commonly used in clinical practice, such as norepinephrine–serotonin reuptake inhibitors, monoamine oxidase inhibitors, norepinephrine–dopamine reuptake inhibitors, tricyclic antidepressants, and selective serotonin reuptake inhibitors. Increasing evidence suggests that curcumin could enhance antidepressant efficacy through several mechanisms of action. With its wide range of pharmacological properties, it is considered a potent antidepressant, as it reduces the inflammatory response [267,268,269,270,271,272,273]; modulates neurotransmitter levels, and inhibits the expression of monoamine oxidase enzymes [17,18,19,20,21]; regulates hypothalamic–pituitary–adrenal (HPA) disorders [274,275,276]; reduces NO [277,278,279,280,281,282]; repairs neurodegeneration and increases neurogenesis and neuronal plasticity, which normally increases BDNF levels [283,284,285,286,287]; regulates mitochondria [288,289,290,291]; and increases antioxidant enzymes [292].

### 4.9. Clinical Trials Performed with Curcumin and Age-Related Disorders

Some clinical trials have devoted attention to investigating the effects of curcumin on ND. These trials are discussed below and are shown in Table 1. The bias risk for each study can be found in Table 2.

A group of healthy elderly people was administered new bioavailable curcumin to analyze its effects on the management of sarcopenia, and it was found that the use of curcumin increased the strength of hand grip and weight lifting strength, increased distance covered before fatigue, and, at the end of the study, improved walking time for a given distance compared to the initial analysis of the study. The small study sample may be a limiting factor, but the randomized controlled pattern, the double-blind possibility, follow-up for a considerable period, and an age range with little variation are superior factors [293].

A study developed by Ghodsi et al. investigated the possible neuroprotective role of curcumin in patients with PD. The scores Unified Parkinson’s disease Rating Scale (MDS-UPDRS) and Parkinson’s Disease Questionnaire (PDQ-39) were evaluated every 3 months until the end of the intervention. Curcumin was shown to be a well-tolerated natural compound but did not significantly enhance the scores applied, presenting no efficiency in ameliorating PD symptoms. Some negative points in this study are the limited number of participants and the sample heterogeneity, which might have masked curcumin’s effects because of the diverse degrees of severity of the disease included. Consequently, the authors suggested that the results could potentially be more significant if the sample consisted of patients with lower disease severity [294].

The effects of curcumin on physical function in moderately functioning older adults with low-grade inflammation were evaluated. Subjects were divided into curcumin C3 Complex^®^, receiving twelve 12 weeks of treatment. The results showed large effect sizes in the short physical performance battery measures of knee extension and flexion peak torque in the curcumin C3 Complex^®^ group but small effect sizes of reductions in galectin-3 and IL-6 inflammatory biomarkers. As a positive factor, curcumin was shown to be safe and well tolerated, and the sample presented high adherence levels (>90%) and retention (94%) during the treatment period. However, the sample size was small, consisting only of Caucasian subjects, the intervention lasted a short period of time, and only one dose of curcumin was administered [228].

Cox et al. investigated the effects of curcumin in a solid lipid form on cognition and mood in a healthy older population. The assessment started with a Mini-Mental State Examination, the Beck Depression Inventory-II, and trait scale of the State-Trait Anxiety Inventory; also, a National Adult Reading Test was applied to measure pre-morbid intellect. Subjects then undertook three rounds of assessment batteries consisting of computerized cognitive tasks that were preceded and followed by an evaluation of state mood. Immediately after the first assessment, a single treatment dose was administered, and then the cognitive tasks were repeated at 1 h and 3 h after the administration. The conclusions were significant enhancement in memory and mood, which included fatigue induced by psychological stress and general fatigue and change in state calmness after chronic treatment, a significant effect on alertness and contentedness after acute and chronic treatment, and reduced total cholesterol and LDL levels. Curcumin was shown to be a safe and well-tolerated compound for the elderly population and even the low applied dose (80 mg) promoted significant and positive results. However, it is important to point out that the sample used was small, and the duration of the intervention was short [295].

Thota et al. [296] examined the effects of administering 180 mg/day of curcumin on the insulin resistance of a group of people at high risk of developing DM2, and it was demonstrated that curcumin showed an improvement in insulin resistance index in the lipid profile, fasting insulin, GSK-3β, and islet amyloid polypeptide, the last two being important markers of AD. The randomized controlled design, control of adherence to interventional treatment, and the possibility of double-blinding are strengths of the study; however, the small sample and short follow-up period are limiting factors.

The study by Rainey-Smith et al. [6] analyzed the use of BCM-95 ^®^ CG (Biocurcumax TM) in men and women with good health and no significant cerebral vascular disease or significant cognitive impairments. No significant difference between the group of intervention and placebo was observed in cognitive function, verbal fluency, mood, perceptual–motor speed, the controlled oral word association test, depression anxiety stress scales, and the Wechsler Digit Symbol Scale of the Wechsler Intelligence Scale for Adults Revised. However, the placebo group demonstrated a decline in function at 6 months, which was not observed in the curcumin group. The study has strengths, such as the randomized controlled design and the long interventional period of 12 months, although visits only occur every 3 months.

The analysis by DiSilvestro et al. investigated the use of 80 mg/day of curcumin for 4 weeks in a population of 19 people, involving healthy men and postmenopausal women, and showed that its use was beneficial in antioxidant activity, due to the increase in catalase, plasma myeloperoxidase, and nitric oxide; in addition to inducing a greater capacity to eliminate free radicals, there was also an improvement in the inflammatory and lipid profile, through reductions in triglycerides and soluble intercellular adhesion molecule (sICAM) [297]. The levels of beta-amyloid protein and alanine aminotransferase were significantly reduced. However, the study has limitations due to the small population sample, short intervention period, and unclear methodology.

Baum et al. studied the effects of using 1 g or 4 g of curcumin for 6 months on the lipid profile of a population with cognitive decline or diagnosed with AD for at least 6 weeks [298]. The study did not show any significant results on the lipid profile. The randomized controlled design and the long follow-up period are important points of the study; however, the small sample is a limiting factor.

A study determined the functional effects of CGM (curcumin–galactomannan) on healthy individuals’ brain waves. Subjects were divided into three groups, and assigned to consume 500 mg of CGM, unformulated curcumin (UC), or placebo capsules twice daily for 30 days, and electroencephalogram (EEG) measurement audiovisual reaction time tests were performed, and a working memory test was performed at baseline and after 30 days. The results indicated that the CGM can influence the brain waves of healthy individuals in a manner consistent with the penetration of the blood–brain barrier, and the electroencephalogram results showed a correlation with the improved audiovisual and working memory tests, contributing to demonstrating the contribution of the CGM in the reduction fatigue and improved memory. The study, however, as it is a pilot study, used a small sample of individuals with a wide age range (35–65 years) [299].

A partial replication study was carried out, with the aim of evaluating similar effects at 4 and 12 weeks of supplementation with Longvida©. Outcome measures included cognitive performance, mood, and biomarkers that were assessed at baseline and after 4 and 12 weeks of treatment, which consisted of Longvida© intake for 12 weeks. The results of the study indicated an improvement in aspects of working memory and lower fatigue scores, and in four weeks, lower scores for anger, tension, confusion, and total mood disturbance. A significant increase in glucose in the group that consumed Longvida© was also observed. The large age range among participants was wide (50 to 85 years old) [300].

Another study evaluated the effects of fish oil and curcumin supplementation on cerebrovascular function in older adults. The results did not show modifications regarding Transcranial Doppler ultrasound, blood pressure, heart rate, arterial compliance, fasting glucose, blood lipids, and C-reactive protein. The authors proposed that since the study of the combined effects of fish oil and curcumin in humans is currently limited, the non-significant effects might be related to the dosages applied and unknown interactions between fish oil and curcumin [301].

Curcumin was used in the form of Theracurmin to analyze its effect on brain amyloid and tau accumulation in adults without dementia. During the intervention, several tests were applied, such as vital signs, electrocardiograms, serum electrolytes, thyroid function, and blood counts, as well as Montreal Cognitive Assessment, Beck Depression Inventory, neuropsychological test battery, and memory functioning questionnaires. The main results included improvement in memory and attention, probably associated with decreases in amyloid and tau accumulation in the brain. One limitation of the article was the small sample size. However, regarding the positive points, the study promoted a relatively long treatment duration and applied sensitive cognitive measures to track memory effects [302].

In Pennsylvania, a study evaluated the effects of a highly absorbent curcumin extract dispersed with colloidal nanoparticles (Theracurmin) in treating adults without dementia on memory performance and its potential impact on neurodegeneration by measuring brain deposition of amyloid plaques and tau tangles. Results showed a significant improvement in attention and memory in adult patients without dementia compared to placebo. PET scan examinations suggested that cognitive and behavioral improvement correlates with reduced accumulation of tau plaques and tangles in regions of the brain that regulate mood and behavior [303].

The effectiveness of curcuminoids as a complement to standard antidepressants in patients with major depressive disorder was investigated. Changes in psychological state based on the Hospital Anxiety and Depression Scale (HADS) and the Beck Depression Inventory II (BDI-II) were used to measure the efficacy of combined curcuminoid–piperine supplementation plus standard therapy. Significant reductions in the HADS total score and depression subscales were greater in the curcuminoid group compared to the control group. Reductions in the BDI-II total score and somatic and cognitive subscale scores were also greater in the curcuminoid group compared to the control group. Although the results are promising, the study was not blinded, suggesting a possible interference with the results [304].

**Table 1:** Clinical trials performed with curcumin on neurodegenerative conditions.

Reference	Model/Country	Population	Intervention/Comparison	Outcomes	Side Effects
Sarcopenia					
[293]	Randomized, placebo-controlled, double-blind clinical trial. India	30 healthy elderly individuals, 13♂, 17♀, 69.8 ± 5.	Participants received 500 mg/day of Cureit or placebo for 3 months.	↑ 1.43% in handgrip strength, a considerable increase of 6.08% in weightlifting strength, and a positive impact on the distance covered before feeling tired (↑ 1.15%, along with speed walking (5.51 m)).	No adverse events were observed.
Parkinson’s disease					
[294]	Pilot, randomized, triple-blind, placebo-controlled, add-on trial. Iran	60 subjects, 45♂, 15♀, 58.2 ± 11.2 y, with idiopathic PD	Subjects received curcumin nanomicelles in capsules 80 mg/day or placebo/9 months. Then, the scores MDS-UPDRS and PDQ-39 were calculated at 3, 6, and 9 months.	Curcumin group did not have a significant improvement in MDS-UPDRS and PDQ-39 scores compared to placebo group.	Nausea, vomiting, and dyspepsia.
Frailty					
[228]	Pilot, 12-week, randomized trial/United States of America	17 subjects, 8♀, 9♂, 66–94 y, moderately functioning and sedentary, with low-grade systemic inflammation.	9 subjects were assigned to Curcumin C3 Complex^®^, receiving 1000 mg/day or placebo. At 0 and at 12 weeks, patients underwent functional testing and lower-limb strength testing. Also, at the beginning of treatment, 4, 8, and 12 weeks, venous blood was collected for safety blood chemistry analyses and biomarkers of inflammation.	Curcumin C3 Complex^®^ group demonstrated large effect sizes in short physical performance battery (d = 0.75), measures of knee extension (d = 0.69), and flexion peak torque (d = 0.82). Furthermore, effects on galectin-3 and IL-6 levels were smaller in curcumin group compared to placebo.	No adverse events were reported.
Dementia					
[295]	Randomized, double-blind, placebo-controlled parallel-group trial. Australia	60 healthy subjects, 22♂, 38♀, 60–85 y.	Subjects were divided into curcumin group (80 m solid lipid formulation (Longvida^®^ Curcumin-400 mg) or placebo/1 timeday/4 weeks. Participants performed 3 sets of computerized cognitive tasks preceded and followed by an evaluation of state mood. After the first set, a single treatment dose was used, and then the assessment was repeated at 1 h and 3 h after dose administration.	The results showed that 1 h after administration, the curcumin group presented significantly enhanced performance on sustained attention and working memory tasks, compared with placebo. Also, working memory and mood were significantly better during chronic treatment (4 weeks). Furthermore, curcumin significantly reduced total cholesterol and LDL cholesterol levels.	No adverse events were reported.
Alzheimer’s disease					
[296]	12-week, 2 × 2 factorial, double-blinded, randomized controlled trial. Australia	29 participants, 12♂, 17♀ (52.3 ± 1.9 y) at high risk of developing diabetes or with impaired fasting glucose	Participants were divided into 4 groups: the placebo; curcumin (2 × 500 mg of curcumin (Meriva^®^), providing 180 mg of curcumin plus 2 × 1000 mg of corn oil/day); ω3, 2 × 1000 mg of fish oil + placebo; or double active (1000 mg of curcumin (Meriva^®^) + 21,000 mg of fish oil.	Curcumin reduced triglyceride levels, fasting insulin, atherogenic index and the HOMA2-IR. There were no significant effects on CRP, TC, HDL-c, LDL-c, fasting glycemia, glycated hemoglobin, and body composition (body weight, muscle mass, body mass index, body fat percentage, circumference waist).	No adverse events were observed.
[305]	Randomized, double-blind, placebo-controlled for 12 months. Australia.	160 healthy individuals; 40–90 y, and no significant cerebral vascular disease; no significant cognitive impairments.	They were randomly assigned to treatment groups with BCM-95 ^®^ CG (Biocurcumax TM) capsule 3 x/day (1500 mg/d) or placebo.	No differences were observed between the placebo and treatment groups in changes in cognitive performance.	Gastrointestinal complaints.
[297]	Prospective randomized, 4 weeks. United States of America	19 healthy participants 17♀, 2 ♂ age 40–60 y	The selected population was assigned to placebo interventions of starch × 80 mg/day of curcumin for 4 weeks	There were no significant effects on TC, LDL-c, HDL-c, superoxide dismutase, and glutathione peroxidase; significant reduction in the levels of TG, intercellular adhesion molecule, and plasma amyloid β protein content. Increased NO, myeloperoxidase, catalase activity, and elimination of free radicals.	No adverse events were observed.
[306]	Randomized, double-blind, placebo-controlled for 6 months. China.	34 individuals 29%♂, 71%♀), aged 73.4 ± 8.8 (progressive decline in memory and cognitive function for at least 6 weeks or diagnosed with AD	They presented 3 groups, one consisting of 10 people (control), the second of 8 people (1 g of curcumin), and the third (4 g of curcumin).	There were no significant effects on the lipid profile (LDL-c, HDL-c, TG, and TC) in both groups receiving curcumin.	Constipation, more, diarrhea, and dizziness.
Cognition					
[299]	Randomized, 30-day, double-blind, placebo-controlled, 3-arm pilot study. India.	18 healthy participants, 12♂ and 6♀, 35–65 y.	Patients were randomized into 3 groups, CGM (500 mg 2×/day for 30 days of curcuma-galactomannoside complex; UC (500 mg 2×/day for 30 days of curcumin with 95% purity) or placebo	CGM: significant ↑↓ in α and β waves, and in the α/β ratio compared to the unformulated curcumin and placebo groups. Furthermore, CGM showed a significant ↓ in audio reaction time (29.8) compared with placebo and 24.6% with UC. Choice-based visual reaction time was also significantly ↓ (36%) in CGM compared to UC and placebo, which yielded 15.36% and 5.2%, respectively.	No adverse events were reported.
[300]	Double-blind, placebo-controlled, 12-week trial/Australia.	79 participants ♀ and ♂healthy, 50–85 y.	Participants were divided into curcumin group (400 mg Longvida© curcumin capsule with 80 mg of curcumin 1×/day/12 weeks) or placebo.	Curcumin group showed better working memory performance at 12 weeks (Serial Threes, Serial Sevens, and performance on a virtual Morris Water Maze) and lower fatigue scores on the POMS at 4 and 12 weeks, and tension, anger, confusion, and total mood disturbance in just 4 weeks.	No adverse events were reported.
[301]	16-week double-blind, randomized placebo-controlled trial/Australia.	152 older sedentary overweight/obese adults, 50–80 y.	Subjects were divided into 4 groups: fish oil + curcumin placebo, curcumin + fish oil placebo, fish oil + curcumin or placebo. Then, patients ingested 6 capsules/day consisting of 2 fish oil capsules and 400 mg Longvida^®^ Optimised Curcumin containing 80 mg of curcumin, or placebo, 2×/d. Then, an evaluation of Transcranial Doppler ultrasound, blood, glycemia, heart rate, arterial compliance, blood lipids, and C-RP was performed.	Curcumin did not significantly affect the performed parameters alone or in combination with fish oil.	Digestive problems and reflux.
[307]	Randomized, double-blind, placebo-controlled pilot clinical trial/USA.	12 participants 9♂ and 3♀ with chronic schizophrenia, 5–51 y.	Patients were randomized into 2 groups: curcumin (180 mg/d) or placebo. A commercially available surface-controlled water-soluble form of 300 mg curcumin (30% formulation: 90 mg pure curcumin) or matching placebo capsules were provided.	Complementary curcumin treatment showed significant improvement in working memory (Z = 2200, *p* = 0.028) and reduced IL-6 levels (Z = 2402, *p* = 0.016) compared to placebo. No significant effect of curcumin on PANSS and Calgary Depression scores was found.	No adverse events were reported.
[302]	18-month, randomized, double-blind, two-group parallel design	40 adults without dementia, 22♀, 18♂ and 50–90 y.	Subjects were divided into placebo group or Theracurmin group (90 mg of curcumin), 2 ×/d/18 months. Depression Inventory and neuropsychological test battery were applied.	Buschke–Fuld Selective Reminding Test presented a consistent long-term retrieval improvement with curcumin (ES = 0.63, *p* = 0.002). Curcumin also improved visual memory and attention.	Transient abdominal pain, gastritis, nausea, and heat.
[303]	Randomized, 18-month, double-blind, placebo-controlled, parallel-group study. EUA	40 participants, 51–84 y, without dementia.	They were randomized into 2 groups: Theracurmin group: 90 mg of curcumin, 2 times d 18 months or placebo group.	Curcumin significantly improved long-term recovery of SRT, visual memory, and attention compared with placebo. Assessment of neurodegeneration using PET scans significantly reduce in the amygdala with curcumin.	No adverse events were reported.
[304]	6-week open study/Tehran, Iran.	111 participants, ♀ and ♂ diagnosed with major depressive disorder	They were divided into standard antidepressant therapy + curcuminoids (1000 mg/d—C3 Complex^®^) or standard antidepressant therapy alone/6 weeks.	Both groups had a reduction in BDI-II total and subscale scores at the end of the study. Significantly greater ↓ in HADS, anxiety, and depression subscales in the curcuminoids versus control group (*p* < 0.001).	Gastrointestinal symptoms

AD: Alzheimer’s disease; BDI-II, Beck Depression Inventory II; CRP: C-reactive protein; HADS: Hospital Anxiety and Depression Scale; HDL-c: high-density lipoprotein; HOMA: homeostatic model for insulin resistance; IL: interleukin; LDL-c: low-density lipoprotein; PANSS: Positive and Negative Symptom Scale; PD: Parkinson’s disease; PMS: Profile of Mood States; TC: total cholesterol. ↓: decrease; ↑: increase.

**Table 2:** Descriptive table of the biases of the included randomized clinical trials.

Study	Question Focus	Allocation Blinding	Double- Blind	Losses (>20%)	Prognostic or Demographic Characteristics	Outcomes	Intention to Treat Analysis	Sample Calculation	Adequate Follow-Up
[293]	Yes	Yes	Yes	No	Yes	Yes	No	Yes	Yes
[294]	Yes	Yes	Yes	Yes	Yes	Yes	Yes	Yes	Yes
[228]	Yes	Yes	Yes	No	Yes	Yes	No	No	Yes
[295]	Yes	Yes	Yes	No	Yes	Yes	No	No	Yes
[296]	Yes	Yes	Yes	No	Yes	Yes	No	Yes	Yes
[305]	Yes	No	Yes	Yes	Yes	Yes	No	Yes	Yes
[297]	No	No	Yes	No	No	Yes	No	No	Yes
[306]	Yes	Yes	Yes	No	Yes	Yes	No	Yes	Yes
[299]	Yes	Yes	Yes	No	Yes	Yes	Yes	Yes	Yes
[300]	Yes	Yes	Yes	Yes	Yes	Yes	Yes	Yes	Yes
[301]	Yes	Yes	Yes	No	Yes	Yes	No	Yes	Yes
[307]	Yes	No	Yes	Yes	Yes	Yes	Yes	Yes	Yes
[302]	Yes	Yes	Yes	No	Yes	Yes	No	No	Yes
[303]	Yes	No	Yes	No	Yes	Yes	Yes	No	Yes
[304]	Yes	No	No	No	Yes	Yes	Yes	Yes	Yes

## 5. Bioavailability and Safety

Curcumin’s bioavailability can be affected by many aspects, such as grinding, drying, and heating processes, and also by the intake of macronutrients, such as dietary lipids, which can interfere with curcumin’s solubility and absorption [308]. Its bioavailability is considered limited due to curcumin’s poor intestinal absorption, high metabolic rate, and fast systemic elimination, contributing to the compound’s low serum levels [309]. However, the development of curcumin and other compound combinations in different formulations enhanced its bioavailability. These formulations include curcumin nanoemulsion, liposomal curcumin, phospholipid curcumin complexes, and even curcumin encapsulation into milk exosomes, which showed higher permeability and bioavailability [20,61,309,310,311,312].

In a study involving male fasting subjects, curcumin was administered in three forms, a completely natural turmeric matrix formulation (CNTMF) and two other commercially available formulations consisting of curcumin with volatile oil and curcumin with phospholipids and cellulose. The analyses showed that the CNTMF form presented the most bioavailability of them [313]. Regarding dosage, in a randomized, double-blind, crossover study, subjects with moderate hyperlipidemia consumed 294 mg of curcuminoids per day in the form of micelles, and this dose was found to be enough to promote accumulation in the blood [314].

Curcumin presents a well-established human safety in the literature [315]. In a randomized and controlled clinical trial involving patients with arthritis, curcumin was administered in doses ranging from 120 to 1500 mg for 4–36 weeks, reduced inflammation and pain levels, and was shown to be a safe treatment method [316]. Also, in a clinical study comparing the administration of curcumin 500 mg (BCM-95^®^) 3 times/day and diclofenac 50 mg, 2 times/day for 28 days in patients with knee osteoarthritis, patients that received curcumin presented similar improvement in the severity of pain, reduction in flatulence episodes, no requirement of H2 blockers, weight reduction, and anti-ulcer effects, proving to be a very safe treatment method presenting a 13% adverse effect rate versus 38% in the diclofenac group (*p* < 0.01) [317].

Furthermore, in chronic kidney disease subjects, supplementation with 500 mg of curcumin tablets, two times/day for six months, reduced plasma pro-inflammatory mediators and lipid peroxidation. During the long-term administration treatment, no serious adverse events were observed, confirming the safety profile of this compound [318]. In addition, in patients with non-alcoholic fatty liver disease, phytosomal curcumin supplementation of 1000 mg/day in two doses for eight weeks was considered safe and well tolerated with no report of severe adverse events during the treatment [319].

## 6. Synthesis and Future Research Endeavors

In the ever-evolving landscape of aging-related disorder research, the exploration of natural compounds as potential therapeutic agents has garnered significant attention. Among these compounds, curcumin has emerged as a promising candidate due to its diverse pharmacological properties and well-documented safety profile. In this section, we delve into the future research directions and endeavors at the intersection of curcumin and aging-related disorders, encompassing a spectrum of scientific inquiries ranging from novel formulations and mechanistic studies to clinical trials and personalized medicine approaches. By elucidating the potential mechanisms of action, optimizing delivery systems, and translating preclinical findings into clinical practice, these endeavors aim to unlock the full therapeutic potential of curcumin in minimizing negative outcomes and improving patient quality of life [13,320,321,322].

### 6.1. Advancing Curcumin Therapy: Exploring Formulations and Unraveling Mechanisms for Aging-Related Disorders

Firstly, researchers must explore novel formulations aimed at enhancing the bioavailability and efficacy of curcumin. This necessitates investigating various delivery systems such as lipid-based nanoparticles, liposomes, or micelles. By delving into the intricacies of these formulations, researchers can unlock new avenues for optimizing the therapeutic potential of curcumin, potentially leading to breakthroughs in its clinical application. Understanding the dynamics of these delivery systems and their interactions with curcumin is crucial for overcoming the challenges associated with its poor solubility and low bioavailability. Through meticulous experimentation and analysis, researchers can pave the way for developing innovative curcumin formulations with enhanced efficacy and therapeutic outcomes.

Scientists also need to dive deeper into conducting thorough mechanistic investigations to gain a deeper understanding of the complex molecular pathways responsible for curcumin’s properties. This involves exploring how curcumin interacts with crucial proteins, signaling pathways, and cellular processes involved in neurodegeneration, cognition, memory, sarcopenia, fragility, and CVD. For instance, they might study more profoundly its effects on proteins like amyloid beta, tau, or BDNF, signaling pathways such as MAPK or NF-κB, and cellular processes like OS or inflammation within the realm of brain metabolism. By unraveling the precise mechanisms through which curcumin operates, researchers can gain profound insights into its therapeutic potential for such disorders. Employing advanced techniques such as proteomics, transcriptomics, and molecular imaging will be instrumental in dissecting the complex interplay between curcumin and various molecular targets within neuronal and other cells. These mechanistic studies are essential for advancing our understanding of curcumin’s neuroprotective properties and guiding the development of targeted therapeutic interventions aimed at combating diseases [323,324].

### 6.2. Unveiling Curcumin’s Therapeutic Potential: Insights from Meticulous Clinical Trials and Advanced Neuroimaging Studies in Neurodegenerative Disorders

In this scenario, the design and implementation of meticulously controlled clinical trials to assess the therapeutic efficacy of curcumin in preventing or treating diverse NDs, such as AD and PD, become of particular interest. By systematically evaluating the effects of curcumin on disease progression, cognitive function, motor symptoms, and quality of life outcomes, researchers can ascertain its true clinical potential in NDs. Moreover, incorporating biomarker assessments and neuroimaging techniques can supply crucial insights into the underlying mechanisms of curcumin’s therapeutic action in the human brain. Through collaborative efforts among clinicians, researchers, and pharmaceutical partners, well-designed clinical trials promise to establish curcumin as an effective and safe therapeutic agent for combating NDs.

In the realm of neuroimaging, researchers must delve more deeply into harnessing advanced neuroimaging techniques, such as functional magnetic resonance imaging (fMRI) and positron emission tomography (PET), to explore the impact of curcumin on both the structural and functional aspects of the brain in individuals afflicted with NDs [325,326]. By employing these sophisticated imaging modalities, researchers can visualize and quantify changes in brain activity, connectivity, and metabolism following curcumin administration. fMRI enables the assessment of dynamic alterations in neuronal activity patterns, while PET offers insights into molecular processes by tracking specific biomarkers associated with neurodegeneration. Through neuroimaging studies, researchers can elucidate how curcumin influences neural networks, neurotransmitter systems, and neuroinflammatory responses implicated in the pathogenesis of NDs. Furthermore, integrating neuroimaging data with clinical outcomes can facilitate the identification of biomarkers for predicting treatment response and monitoring disease progression [327,328,329].

### 6.3. Unlocking Synergistic Therapeutic Strategies: Exploring Curcumin Combinations and Molecular Interactions in Disease Management

Researchers should also explore the potential synergistic effects of curcumin associated with other natural compounds, pharmaceutical agents, or lifestyle interventions for managing aging-related diseases. Investigating these combination therapies offers a multifaceted approach to treatment, leveraging the complementary mechanisms of action of different compounds or interventions. Researchers can uncover novel strategies to enhance therapeutic outcomes while minimizing adverse effects by examining how curcumin interacts with other substances or interventions. Furthermore, exploring combination therapies underscores the importance of integrative approaches to healthcare, where traditional medicine intersects with modern pharmacology and lifestyle modifications. Through rigorous experimentation and clinical trials, researchers can elucidate the optimal combinations, dosages, and treatment regimens that maximize the therapeutic potential of curcumin in synergy with other interventions, ultimately offering hope for more effective management of NDs.

Additionally, future investigations could delve into elucidating the dynamic interplay between curcumin treatment and the intricate molecular networks underlying aging-related diseases employing cutting-edge bioinformatics tools. Using advanced network analysis and pathway modeling, researchers can explore the temporal and spatial effects of curcumin on multi-omics data. Additionally, integrating emerging technologies like single-cell sequencing could provide unprecedented insights into cell-specific responses to curcumin therapy. This comprehensive approach promises to uncover novel biomarkers and therapeutic targets essential for developing personalized interventions against NDs [330].

### 6.4. Fostering Collaboration for Curcumin Translation: Bridging Academia, Industry, Regulation, and Healthcare for Age-Related Disease Management

Efforts to apply promising preclinical findings into daily clinical practice necessitate collaborative endeavors among academia, industry, regulatory agencies, and healthcare providers [331,332]. By fostering robust partnerships, stakeholders can navigate regulatory frameworks, streamline clinical trials, and ensure curcumin-based interventions’ safe and effective implementation. Additionally, considering the cost-effectiveness of these interventions is crucial for widespread adoption. Balancing efficacy with affordability, this multidisciplinary approach aims to optimize therapeutic outcomes while minimizing economic burdens on patients and healthcare systems. By emphasizing patient-centered care, this strategy aims to enhance the quality of life for individuals afflicted by these debilitating conditions, ensuring equitable and sustainable access to innovative treatments.

### 6.5. Unraveling the Genetic Basis of Curcumin Response: Genome-Wide Association Studies in Aging-Related Disease Management

Genome-Wide Association Studies (GWASs) present a promising avenue for unraveling the genetic underpinnings of response to curcumin treatment in aging-related diseases such as NDs. By analyzing large cohorts of patients with diverse genetic backgrounds, GWAS can identify genetic variants associated with differential treatment outcomes, including variations in treatment efficacy and susceptibility to adverse effects [333]. Additionally, GWAS can elucidate gene–drug interactions that modulate curcumin metabolism, target engagement, and downstream biological responses.

A comprehensive GWAS in this realm could involve genotyping thousands of individuals with NDs who have undergone curcumin treatment alongside appropriate control groups. Integrating multi-omics data, such as genomic, transcriptomic, and epigenomic profiles, with clinical parameters and treatment responses can provide a holistic understanding of curcumin therapy’s genetic architecture. Furthermore, leveraging advanced bioinformatics and systems biology approaches, such as pathway enrichment analysis and network modeling, can uncover key biological pathways and candidate genes implicated in curcumin-mediated neuroprotection. These findings could inform the development of personalized treatment strategies and facilitate the identification of novel therapeutic targets for intervention.

However, conducting GWASs in this context poses several challenges, including the need for large, well-characterized patient cohorts, rigorous quality control measures to minimize confounding factors, and robust statistical methodologies to account for genetic heterogeneity and population stratification. Additionally, ethical considerations surrounding data privacy, consent, and equitable access to benefits necessitate careful deliberation and adherence to ethical guidelines throughout the study. Despite these challenges, GWASs hold immense potential to speed up the translation of preclinical findings into clinical practice, paving the way for precision medicine approaches tailored to individual genetic profiles and optimizing therapeutic outcomes for patients with NDs undergoing curcumin treatment [334].

## 7. Conclusions

This systematic review underscores the promising potential of curcumin as a natural compound with diverse therapeutic effects in combating age-related disorders. By targeting key pathways involved in inflammation, OS, and mitochondrial dysfunction, curcumin demonstrates significant benefits in improving cognitive function, reducing neurodegeneration, and enhancing muscle health in the elderly population. The review highlights the importance of further research to elucidate the specific mechanisms of action of curcumin and optimize its dosage and formulation for maximum efficacy. Standardized protocols and well-designed clinical trials are essential to validate the findings and establish curcumin as a safe and effective intervention for promoting healthy aging and preventing age-related conditions. Collaborative efforts among researchers, clinicians, and pharmaceutical partners are crucial in advancing our understanding of curcumin’s therapeutic potential and translating this knowledge into clinical practice. Overall, the evidence presented in this review supports the notion that curcumin holds promise as a valuable adjunct in the management of age-related disorders, offering a natural and potentially effective approach to enhancing the quality of life in aging individuals.

## Abbreviations

AChE	acetylcholinesterase
AD	Alzheimer’s disease
AMPK	adenosine 5′-monophosphate-activated protein kinase
APP	amyloid precursor protein
APPsw	APP Swedish Mutant
Bax	Bcl-2-associated protein X
Bcl-2	B-cell lymphoma 2
Cdk5	cyclin-dependent kinase 5
CNTMF	completely natural turmeric matrix formulation
COX-2	cyclooxygenase 2
DM2	diabetes mellitus type 2
DNA	deoxyribonucleic acid
FeONPs-Cur	iron oxide nanoparticles capped with curcumin
GSK-3β	glycogen synthase kinase-3β
HO-1	heme oxygenase-1
IL	interleukin
JAK/STAT	Janus kinase/signal transducer and activator of transcription
JNK	c-Jun N-terminal kinase
Keap1	Kelch-like ECH-associated protein 1
MCT2	monocarboxylate transporter 2
mTOR	mammalian target of rapamycin
NADPH	nicotinamide adenine dinucleotide phosphate
NDs	neurodegenerative diseases
NF-κβ	nuclear factor-kappabeta
NO	nitric oxide
Nrf2	nuclear factor erythroid 2-related factor 2
OS	oxidative stress
PD	Parkinson’s disease
PI3K	phosphoinositide 3-kinases
PI3K/AKT	phosphatidylinositol 3-kinase/protein kinase B
PPARγ	peroxisome proliferator-activated receptor gamma
RAGE	glycation end products
RNS	reactive nitrogen species
ROS	oxygen species
p-	phosphorylated
PPAR-γ	peroxisome proliferator-activated receptor gamma
Th17	T helper 17
TNF	tumor necrosis factor
TNF-α	tumor necrosis factor-alpha
TLR	Toll-like receptor
